# Obesity and Impaired Male Fertility: An Association of Clinical Importance

**DOI:** 10.7759/cureus.110987

**Published:** 2026-06-16

**Authors:** Kholoud Krimi, El-Houcine Sebbar, Mohammed Choukri

**Affiliations:** 1 Central Laboratory Department, Laboratory of Biochemistry, Mohammed VI University Hospital, Faculty of Medicine and Pharmacy, Mohammed First University, Oujda, MAR

**Keywords:** fertility, impaired male fertility, male fertility, obesity, obesity and fertility

## Abstract

Background: Male infertility is a major contributor to couple infertility, and increasing evidence suggests that excess body weight may adversely affect semen quality. Obesity and overweight have been associated with hormonal imbalance, oxidative stress, and metabolic disturbances that may impair spermatogenesis. The aim of this study was to evaluate the association between body mass index (BMI) and semen abnormalities in infertile men.

Methods: We conducted a retrospective descriptive study over a period of two years, including 112 men who consulted for infertility at the Medically Assisted Reproduction Center of Mohammed VI University Hospital, Oujda, Morocco. Patients were classified into three BMI groups: normal BMI, overweight, and obese. Semen analysis was assessed according to World Health Organization criteria. Quantitative and qualitative semen abnormalities were recorded and compared across BMI groups. Statistical analysis was mainly performed using the chi-square test.

Results: Among the 112 patients, 40 (35.7%) had a normal BMI, 52 (46.4%) were overweight, and 20 (17.9%) were obese. Normal semen parameters were more frequent in patients with normal BMI than in overweight and obese patients. Abnormal semen analyses were observed in 24 of 40 normal-BMI patients (60.0%), 46 of 52 overweight patients (88.5%), and 16 of 20 obese patients (80.0%). There was a statistically significant association between BMI category and semen analysis status (𝜒^2^ = 10.42, p = 0.005). Pairwise analysis showed that the main difference was between normal-BMI and overweight patients (p = 0.003). Azoospermia was more frequent in overweight and obese patients than in normal-weight patients (21.2% and 25.0% vs. 15.0%), although this difference was not statistically significant (p = 0.611). Combined and more severe semen abnormalities were descriptively more frequent in patients with elevated BMI.

Conclusion: Excess body weight was associated with a higher frequency of abnormal semen parameters in this cohort of infertile men. Overweight and obese patients showed a greater burden of combined and severe semen abnormalities compared with patients with normal BMI. These findings support the role of BMI as a relevant clinical factor in the evaluation of male infertility and suggest that weight control may represent an important component of fertility-oriented management. Larger studies are needed to confirm these results and better define the relationship between overweight, obesity, and specific semen abnormalities.

## Introduction

Infertility affects approximately 15% of couples worldwide, with male factors contributing to nearly half of all cases, most commonly through abnormalities in sperm parameters [1]. Obese men frequently exhibit reduced fertility in parallel with lower testosterone levels [2]. In this context, hypogonadism has been associated with testosterone deficiency, impaired spermatogenesis, and metabolic disorders such as obesity [3]. Moreover, obesity may directly alter sperm quality through several mechanisms, including insulin resistance, chronic inflammation, and oxidative stress [4]. The detrimental effect of obesity on semen quality also appears to vary according to the degree of obesity [5]. At the same time, the global rise in overweight and obesity has raised increasing concern regarding their impact on male reproductive health. Excess body weight may impair fertility through hormonal disturbances, oxidative stress, and an altered testicular microenvironment. In particular, increasing body mass index (BMI) has been associated with a higher frequency and greater complexity of sperm abnormalities [5]. These observations are consistent with the existing literature and support the deleterious effect of overweight and obesity on spermatogenesis. Obesity may therefore be considered a potentially modifiable risk factor for male infertility. In this context, the present study highlights the association between excess body weight and impaired sperm quality, emphasizing that BMI assessment should be integrated into the routine diagnostic evaluation of male infertility and that targeted nutritional and metabolic management may be beneficial.

## Materials and methods

Study design

This is a retrospective descriptive study conducted at the Medically Assisted Reproduction Center of Mohammed VI University Hospital, Oujda, Morocco.

Study population

The study included 112 medical records of patients evaluated for male infertility over a two-year period, between May 2023 and May 2025.

Inclusion criteria

Patients who sought a consultation for male infertility, with a complete medical record including anthropometric data, particularly BMI, and a recent semen analysis (spermogram and spermocytogram) performed according to the World Health Organization (WHO) recommendations [6].

Exclusion criteria

**Figure 1:**
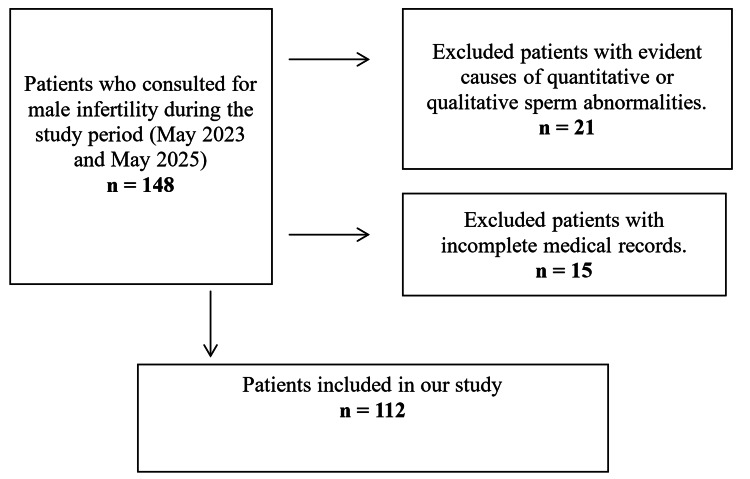
The flowchart of patient selection, including inclusion, and exclusion criteria

Patients with known testicular pathology, such as severe varicocele, a history of chemotherapy, untreated cryptorchidism, or other major conditions likely to affect semen quality, were excluded from the analysis. Patients with incomplete medical records were also excluded from the study (Figure 1).

Variables analyzed

For each patient, the following data were collected: age, weight, and height, which were used to calculate BMI according to the formula: $\mathrm{BMI}=\frac{\text{weight (kg)}}{\text{height (m)}^{2}}$. Patients were then classified into three groups according to BMI: normal BMI: 18.5-24.9 kg/m², overweight: 25.0-29.9 kg/m², and obesity: ≥30 kg/m². Semen analyses, including spermogram and spermocytogram, were performed in the center’s laboratory in accordance with WHO standards. The following semen parameters were evaluated: semen volume, sperm concentration, progressive and total motility, sperm morphology, and sperm vitality. Semen abnormalities were classified as follows [6]: oligospermia as a sperm concentration <15 million/mL, asthenospermia as progressive motility <32%, teratospermia as normal morphology <4%, necrospermia as vitality <58%, azoospermia as the complete absence of spermatozoa, cryptozoospermia as spermatozoa detectable only after centrifugation, hypospermia as a semen volume <1.5 mL, and aspermia as the complete absence of ejaculate.

Statistical analysis

Data were entered and analyzed using Microsoft Excel 2019 (Microsoft Corporation, Redmond, WA). Descriptive results were expressed as numbers and percentages. A comparison between the groups was performed mainly using the chi-square (χ²) statistical analysis.

## Results

Study population

We analyzed the medical records of 112 patients who consulted for male infertility at the Medically Assisted Reproduction Center of Mohammed VI University Hospital, Oujda, Morocco.

BMI distribution

Patients were aged between 28 and 44 years, with a mean age of 35.6 years. They were classified according to their BMI. Among the study population, 40 patients (35.71%) had a normal BMI, 52 (46.43%) were overweight, and 20 (17.85%) had a BMI >30 kg/m², corresponding to obesity. The mean BMI was 22.2 ± 1.5 kg/m² in the normal-weight group, 27.1 ± 1.4 kg/m² in the overweight group, and 34.4 ± 3.0 kg/m² in the obesity group.

Semen parameters in patients with normal BMI

Among patients with a normal BMI, 16 (40%) had a normal semen analysis (spermogram and spermocytogram), whereas 24 (60%) had abnormal semen parameters. The abnormalities were distributed as follows: nine patients (22.5%) had oligoasthenospermia, six (15%) had oligoasthenonecrospermia, six (15%) had isolated azoospermia, one (2.5%) had isolated asthenospermia, and two (5%) had isolated oligospermia (Table 1).

**Table 1:** Sperm anomalies detected in normal BMI group (n = 40) BMI: body mass index

Type of abnormality	n (%)
Oligospermia + asthenospermia	9 (22.5%)
Oligospermia + asthenospermia + necrospermia	6 (15%)
Azoospermia	6 (15%)
Oligospermia	2 (2.5%)
Asthenospermia	1 (5%)

Semen parameters in overweight patients

Among overweight patients, six (11.5%) had a normal semen analysis, whereas 46 (88.5%) had abnormal semen parameters. These abnormalities were distributed as follows: 14 (26.9%) cases of oligoasthenonecrospermia associated with teratospermia, 11 (21.2%) cases of azoospermia, six (11.5%) cases of oligoasthenonecrospermia, four (7.7%) cases of oligoasthenospermia, four (7.7%) cases of isolated asthenospermia, two (3.8%) cases of hypospermia, two (3.8%) cases of aspermia, one (1.9%) case of cryptozoospermia, one (1.9%) case of isolated oligospermia, and one (1.9%) case of asthenospermia associated with necrospermia (Table 2).

**Table 2:** Sperm anomalies detected in overweight group (n = 52)

Type of abnormality	n (%)
Oligospermia + asthenospermia + necrospermia + teratospermia	14 (26.9%)
Azoospermia	11 (21.2%)
Oligospermia + asthenospermia + necrospermia	6 (11.5%)
Oligospermia + asthenospermia	4 (7.7%)
Asthenospermia	4 (7.7%)
Hypospermia	2 (3.8%)
Aspermia	2 (3.8%)
Cryptozoospermia	1 (1.9%)
Oligozoospermia	1 (1.9%)
Asthenospermia + necrospermia	1 (1.9%)

Semen parameters in obese patients

Among obese patients, four (20%) had a normal semen analysis, whereas 16 (80%) had abnormal semen parameters. The abnormalities were distributed as follows: five (25%) cases of azoospermia, five (25%) cases of oligoasthenospermia associated with hypospermia, three (15%) cases of oligoasthenonecrospermia associated with teratospermia, one (5%) case of isolated asthenospermia, one (5%) case of asthenospermia associated with necrospermia, and one (5%) case of hypospermia with retrograde ejaculation associated with oligospermia, asthenospermia, and teratospermia in a patient with a BMI of 47.8 kg/m² (Table 3).

**Table 3:** Sperm anomalies detected in obese group (n = 20)

Type of abnormality	n (%)
Azoospermia	5 (25%)
Oligospermia + asthenospermia + hypospermia	5 (25%)
Oligospermia + asthenospermia + necrospermia + teratospermia	3 (15%)
Asthenospermia	1 (5%)
Asthenospermia + necrospermia	1 (5%)
hypospermia + retrograde ejaculation + oligospermia + asthenospermia + teratospermia	1 (5%)

Normal semen parameters were more frequent in patients with a normal BMI (16, 40%) than in those who were overweight (6, 11.5%) or obese (4, 20%). In contrast, abnormal semen profiles, particularly combined abnormalities involving sperm count, motility, vitality, and morphology, were more frequent in patients with elevated BMI. A chi-square test compares normal and abnormal semen analysis across the three BMI groups (χ² = 10.42, p = 0.005) (Table 4). There is a statistically significant association between BMI category and semen analysis status. Abnormal semen parameters were more frequent in overweight and obese patients than in patients with normal BMI, with the highest proportion observed in the overweight group.

**Table 4:** Chi-square test comparing normal and abnormal semen analysis across the three groups BMI: body mass index

BMI group	Total patients	Normal semen analysis, n (%)	Abnormal semen analysis, n (%)	χ²	p
Normal BMI	40	16 (40.0%)	24 (60.0%)	10.42	0.005
Overweight	52	6 (11.5%)	46 (88.5%)		
Obese	20	4 (20.0%)	16 (80.0%)		
Total	112	26 (23.2%)	86 (76.8%)		

The main difference was observed between patients with normal BMI and those with overweight. The difference between normal BMI and obese patients did not reach statistical significance, likely in part due to the smaller sample size among obese patients (Table 5).

**Table 5:** Statistical analysis of abnormal sperm among the three groups BMI: body mass index

Comparison	p value
Normal BMI vs. overweight	0.003
Normal BMI vs. obese	0.154
Overweight vs. obese	0.449

Chi-square test for azoospermia across BMI groups (χ² = 0.99, p = 0.611) was performed. Although azoospermia appeared more frequent in overweight and obese patients, the difference was not statistically significant (Table 6).

**Table 6:** Frequency of azoospermia among the three groups BMI: body mass index

BMI group	Azoospermia, n (%)	No azoospermia, n (%)	χ²	p
Normal BMI	6 (15.0%)	34 (85.0%)	0.99	0.611
Overweight	11 (21.2%)	41 (78.8%)		
Obese	5 (25.0%)	15 (75.0%)		
Total	22 (19.6%)	90 (80.4%)		

The proportion of abnormal semen analyses differed significantly across groups. Abnormal semen parameters were observed in 24 of 40 patients with normal BMI (60.0%), 46 of 52 overweight patients (88.5%), and 16 of 20 obese patients (80.0%), with a statistically significant association between BMI category and semen analysis status (χ² = 10.42, p = 0.005). Pairwise analysis showed a significant difference between normal-BMI and overweight patients (p = 0.003), whereas the comparisons between normal-BMI and obese patients, and between overweight and obese patients, were not statistically significant. Azoospermia was more frequent in overweight and obese patients than in normal-weight patients (21.2% and 25.0% vs. 15.0%), although this difference was not statistically significant (p = 0.611). Combined and more severe sperm abnormalities were more commonly observed in overweight and obese patients.

However, these findings should be interpreted with caution, given the relatively limited sample size, particularly in the obese subgroup, which may have reduced the statistical power to detect differences for some individual semen abnormalities. Larger studies including a greater number of patients and more balanced BMI subgroups are needed to confirm these results, better clarify the relationship between excess body weight and semen quality, and determine whether specific semen alterations are more strongly associated with overweight or obesity.

## Discussion

Body weight and adiposity can influence overall metabolism [7,8]. Metabolic irregularities may impair spermatogenesis and have been linked to reduced sperm production [9]. The effect of age on male fertility remains controversial, with some studies showing a link between advancing age and impaired spermatogenesis, whereas others found no clear interaction between these two parameters [10].

In a study by Liu et al., a significant difference was found in 8 of 14 body composition indicators after controlling for age, suggesting that age may mask the relationship between body composition and sperm parameters. That study also suggested that abnormal increases in weight may contribute to azoospermia [11].

In our series of 112 infertile men, abnormal semen parameters were significantly more frequent in patients with elevated BMI than in those with normal BMI. Abnormal semen analyses were observed in 24 of 40 normal-BMI patients (60.0%), 46 of 52 overweight patients (88.5%), and 16 of 20 obese patients (80.0%), with a statistically significant association between BMI category and semen analysis status (χ² = 10.42, p = 0.005). Pairwise analysis showed that the difference was mainly driven by the comparison between normal-BMI and overweight patients (p = 0.003), whereas comparisons involving obese patients did not reach statistical significance, likely in part due to the smaller sample size of the obese subgroup. These findings support the hypothesis that excess body weight is associated with impaired semen quality, even though the relationship may not be strictly linear across BMI categories.

A study by Ma et al. showed that obesity was associated with poorer sperm quality, including reduced sperm vitality, impaired motility, and morphological abnormalities, and concluded that sperm dynamics and morphology were significantly worse in the obesity group. However, no statistically significant difference was reported in total sperm count between obese and nonobese groups [4]. Our results are in line with these observations, as combined and more severe semen abnormalities were more frequently observed in overweight and obese patients, particularly mixed alterations involving sperm count, motility, vitality, and morphology. In our study, overweight patients had the highest proportion of abnormal semen profiles, and severe combined abnormalities such as oligoasthenonecrospermia associated with teratospermia were especially frequent in men with elevated BMI.

In a study evaluating the association between BMI and sperm quality in 4,860 semen samples, Luque et al. demonstrated that sperm concentration, sperm count, and motility were significantly reduced in underweight and morbidly obese men (BMI ≥40 kg/m²) compared with normal-weight, overweight, and obese men. They also found that oligospermia and teratospermia were more frequent in morbidly obese men than in the other BMI groups [5]. Their study highlighted not only the negative effects of obesity on sperm quality but also the impact of underweight on fertility, and showed that sperm concentration and sperm count were among the semen parameters most affected by BMI extremes [5]. In our study, although underweight patients were not represented, we similarly found that abnormal semen profiles were more common in men with increased BMI. Moreover, azoospermia was descriptively more frequent in overweight and obese patients than in normal-weight patients (21.2% and 25.0% vs. 15.0%), although this difference did not reach statistical significance (p = 0.611). This suggests that while increased BMI may be associated with more severe alterations in spermatogenesis, our sample size may have been insufficient to detect statistically significant differences for specific abnormalities such as azoospermia.

These effects on spermatogenesis and fertility may be related to hormonal imbalance, chronic inflammation, and oxidative stress; additionally, excess adipose tissue may lower testosterone levels and promote sperm DNA damage through oxidative stress [12,13]. Testosterone deficiency may in turn promote fat accumulation, creating a vicious cycle between obesity, androgen deficiency, and impaired reproductive function [4]. Increased BMI has also been associated with reduced sperm mitochondrial activity and increased DNA fragmentation [14].

Our findings are consistent with these pathophysiological hypotheses. Although we did not assess hormonal or oxidative stress markers directly, the higher frequency of abnormal semen profiles and the predominance of combined abnormalities in overweight and obese patients support the concept that excess body weight may adversely affect several aspects of sperm quality simultaneously rather than a single isolated parameter. The fact that the biggest difference in our cohort was observed between normal-BMI and overweight patients may suggest that semen quality impairment can already become apparent at the overweight stage, before the development of more severe obesity.

These findings support the idea that maintaining a healthier body composition may be important for preserving fertility. Weight control is therefore increasingly advised in public health strategies [11,15]. Moderate exercise and a healthy diet may improve sperm quality and fertility [16]. In our study, the significantly lower proportion of normal semen analyses among overweight and obese men compared with normal-BMI patients further supports the potential reproductive benefit of weight management.

However, our results should be interpreted with caution because of the relatively limited sample size, particularly in the obese subgroup, which may have reduced the statistical power of some subgroup comparisons. Larger studies including a greater number of patients and more balanced BMI categories are needed to confirm these findings, better define the relationship between overweight, obesity, and semen quality, and determine whether specific sperm abnormalities are more strongly associated with particular BMI categories.

Limitations of the study

This study has several limitations, including its retrospective, single-center design and relatively limited sample size, particularly in the obese subgroup, which may have reduced statistical power. In addition, potential confounding factors such as lifestyle habits, metabolic disorders, hormonal status, and fat distribution were not assessed.

## Conclusions

This study showed a significant association between BMI category and semen analysis status in infertile men. Abnormal semen parameters were more frequent in overweight and obese patients than in those with normal BMI, with the highest proportion observed in the overweight group. In addition, combined and more severe semen abnormalities were more commonly found in patients with elevated BMI, supporting the hypothesis that excess body weight may negatively affect several components of sperm quality simultaneously. Although azoospermia was more frequent in overweight and obese men, the difference did not reach statistical significance, likely because of the limited sample size, particularly in the obese subgroup. Nevertheless, the overall pattern of our results is consistent with the growing body of evidence linking excess body weight to impaired male reproductive function. Obesity should therefore be regarded as a modifiable risk factor for male infertility. Weight optimization through nutritional, metabolic, and lifestyle interventions may have potential benefits for reproductive health. Further studies with larger and more balanced cohorts are needed to confirm these findings, clarify the mechanisms involved, and determine which semen abnormalities are most strongly associated with overweight and obesity.
